# Basal-like phenotype is not associated with patient survival in estrogen-receptor-negative breast cancers

**DOI:** 10.1186/bcr1649

**Published:** 2007-01-31

**Authors:** Mervi Jumppanen, Sofia Gruvberger-Saal, Päivikki Kauraniemi, Minna Tanner, Pär-Ola Bendahl, Mikael Lundin, Morten Krogh, Pasi Kataja, Åke Borg, Mårten Fernö, Jorma Isola

**Affiliations:** 1 Department of Pathology, Seinäjoki Central Hospital, Hanneksenrinne 7, FIN-60220 Seinäjoki, Finland; 2 Institute of Medical Technology, University and University Hospital of Tampere, Biokatu 6, FIN-33520 Tampere, Finland; 3 Department of Oncology, Clinical Sciences, University of Lund, Klinikgatan 7, SE-221 85 Lund, Sweden; 4 Department of Oncology, Tampere University Hospital, Teiskontie 35, FIN-33520 Tampere, Finland; 5 Biomedical Informatics group, Department of Oncology, University of Helsinki, Haartmanninkatu 8, FIN-00290 Helsinki, Finland; 6 Department of Theoretical Physics, Lund University, Sölvegatan 14A, SE-221 85 Lund, Sweden

## Abstract

**Introduction:**

Basal-phenotype or basal-like breast cancers are characterized by basal epithelium cytokeratin (CK5/14/17) expression, negative estrogen receptor (ER) status and distinct gene expression signature. We studied the clinical and biological features of the basal-phenotype tumors determined by immunohistochemistry (IHC) and cDNA microarrays especially within the ER-negative subgroup.

**Methods:**

IHC was used to evaluate the CK5/14 status of 445 stage II breast cancers. The gene expression signature of the CK5/14 immunopositive tumors was investigated within a subset (100) of the breast tumors (including 50 ER-negative tumors) with a cDNA microarray. Survival for basal-phenotype tumors as determined by CK5/14 IHC and gene expression signature was assessed.

**Results:**

From the 375 analyzable tumor specimens, 48 (13%) were immunohistochemically positive for CK5/14. We found adverse distant disease-free survival for the CK5/14-positive tumors during the first years (3 years hazard ratio (HR) 2.23, 95% confidence interval (CI) 1.17 to 4.24, *p *= 0.01; 5 years HR 1.80, 95% CI 1.02 to 3.15, *p *= 0.04) but the significance was lost at the end of the follow-up period (10 years HR 1.43, 95% CI 0.84 to 2.43, *p *= 0.19). Gene expression profiles of immunohistochemically determined CK5/14-positive tumors within the ER-negative tumor group implicated 1,713 differently expressed genes (*p *< 0.05). Hierarchical clustering analysis with the top 500 of these genes formed one basal-like and a non-basal-like cluster also within the ER-negative tumor entity. A highly concordant classification could be constructed with a published gene set (Sorlie's intrinsic gene set, concordance 90%). Both gene sets identified a basal-like cluster that included most of the CK5/14-positive tumors, but also immunohistochemically CK5/14-negative tumors. Within the ER-negative tumor entity there was no survival difference between the non-basal and basal-like tumors as identified by immunohistochemical or gene-expression-based classification.

**Conclusion:**

Basal cytokeratin-positive tumors have a biologically distinct gene expression signature from other ER-negative tumors. Even if basal cytokeratin expression predicts early relapse among non-selected tumors, the clinical outcome of basal tumors is similar to non-basal ER-negative tumors. Immunohistochemically basal cytokeratin-positive tumors almost always belong to the basal-like gene expression profile, but this cluster also includes few basal cytokeratin-negative tumors.

## Introduction

cDNA microarray studies have shown that the most powerful denominator in determining the gene expression profiles and prognostic groups of breast cancer is estrogen receptor (ER) and ER-related genes [1-5]. Breast cancers have been separated by gene expression profiles into luminal, basal-like, ERBB2, and normal breast-like subgroups [6-9]. Basal-like tumors express many of the genes characteristic of breast basal epithelial cells [6] and the most typical feature of basal-like breast cancers is the lack of expression of ER and genes usually co-expressed with ER [6-9].

In addition to the gene expression microarray studies, basal-phenotype breast tumors have long been identified by using basal cytokeratin immunohistochemistry (IHC) [10-20]. Basal cytokeratin (CK5/14/17)-positive tumors represent about 10% of sporadic breast carcinomas and are almost exclusively ER-negative, poorly differentiated, and associated with epidermal growth factor receptor (EGFR), p53, vimentin, and c-*kit *immunopositivity and Bcl-2 negativity [11,12,14-16,19-21]. Even though gene expression studies separate the basal-like tumors from the ERBB2 tumor subgroup [6-9], there are some immunohistochemically basal cytokeratin-expressing tumors that show *HER-2 *oncogene amplification [12,17,22]. The relationship between immunohistochemical and microarray-based classification of basal-phenotype breast cancer has not been established.

Apart from hypothesis-generating scientific research, a breast tumor classification should correlate with the clinical outcome of patients or predict efficacy to therapy. Negative ER status, which is the most prominent feature of basal-phenotype tumors, is a well-established prognostic and predictive factor in breast cancer. Microarray studies have shown that basal-like tumors have poor prognosis when compared with ER-positive luminal tumor groups but not when compared with a ERBB2 tumor cluster [7,8]. Immunohistochemical studies with basal cytokeratin IHC for the basal breast cancer phenotype classification have almost exclusively addressed the fact that basal-phenotype tumors have poor prognosis, but they have also made the comparison in cohorts not selected by matching ER status (ER-negative) [10,11,16,17,20,23-25]. In this study we defined the gene expression profile of basal cytokeratin immunopositive tumors and studied the clinical outcome especially within the ER-negative tumor entity.

## Materials and methods

### Tumor samples

The tumor cohort comprised 445 primary stage II breast cancers collected from the South Sweden Health Care Region between 1985 and 1994 with approval from the Lund University Hospital ethics committee; the cohort was described previously in more detail by Chebil and colleagues [26]. In the present study, patients treated with 20 mg of tamoxifen daily for 2 years with complete follow-up data and uniform immunohistochemical method for hormone receptor analysis were included. Radical mastectomy or breast-conserving surgery was used with axillary lymph node dissection. Radiotherapy was introduced for all patients treated with breast-conserving surgery and for patients with lymph-node-positive disease. The patients were not treated with adjuvant chemotherapy. The median follow-up time for distant disease-free survival was 6 years.

### Immunohistochemistry

The formalin-fixed paraffin-embedded sample material was provided as eight tissue microarrays (TMAs) containing three core samples (diameter 0.6 mm) for each primary tumor. Immunohistochemical staining with CK5/CK14/p63 antibody cocktail (XM26, dilution 1:400, Novocastra, Newcastle upon Tyne, UK; LL002, dilution 1:400, Novocastra; 4A4+Y4A3, dilution 1:1,500, Neomarkers, Fremont, CA, USA, respectively) and with p53 antibody (DO-7, dilution 1:500, Novocastra) was performed as described previously [12,22]. Hormone receptors (ER and progesterone receptor) were conducted earlier by IHC from the original tissue blocks as described by Chebil and colleagues [26].

Analysis of the *HER-2 *oncogene amplification was conducted by using a chromogenic *in situ *hybridization (CISH) method as described previously [27]. The histological type of the tumors was determined in accordance with the WHO classification as described by Chebil and colleagues [26].

### Sample scoring

Immunohistochemically stained TMA samples for CK5/CK14/p63 and p53 as well as *HER-2 *CISH stainings were scanned with a virtual microscopy technique as described previously [28]. Immunostaining for CK5/CK14/p63 was considered CK5/14-positive if at least 20% of the tumor cells showed cytoplasmic staining and positive for p63 when the staining was nuclear. p53 was regarded as positive when at least 20% of the tumor cells were stained. *HER-2 *oncogene was considered amplified if six or more gene copies were found per cell in at least 10% of the tumor cells.

### Statistical analysis

Fisher's exact test and the χ^2 ^test were used to test the significance of the cross-tabulated data (using Stata 9.2 (Stata Corporation, College Station, TX, USA) and MedCalc (MedCalc Software, Mariakerke, Belgium) statistical software packages). Survival analyses were calculated with Kaplan-Maier life table curves, a log-rank test and a univariate Cox model. Distant disease-free survival was calculated from the primary diagnosis to the date of an event (distant recurrence or death) or, for event-free patients, to the date of the most recent follow-up. All reported *p *values are two-sided.

### Gene expression microarrays

cDNA microrrays were manufactured in the SWEGENE Microarray Facility, Department of Oncology, Lund University. The gene set consisted of 24,301 sequence-verified IMAGE clones (Research Genetics, Huntsville, AL, USA) and 1,296 internally generated clones, together representing about 16,000 Unigene clusters (build 180) and about 1,200 unclustered expressed sequence tags. The clones were amplified by polymerase chain reaction with vector-specific primers essentially as described previously [29].

A selected subset (*n *= 100, of which 50 were ER-negative) from the total cohort was analyzed with microarrays. Nineteen of these tumors showed positive CK5/14 staining and the rest were negative. Only one of the CK5/14-positive tumors was ER-positive. Total RNA was extracted from grossly dissected frozen tissue samples (about 100 mg) by the subsequent use of Trizol (Invitrogen, Carlsbad, CA, USA) and the RNeasy kit (Qiagen, Hilden, Germany). For each hybridization, 15 μg of Universal Human Reference RNA (Stratagene, La Jolla, CA, USA) was used to synthesize reference Cy5-labeled targets and 25 μg of sample total RNA for Cy3-labeled targets. Anchored oligo(dT) primers, the CyScribe indirect amino-allyl cDNA synthesis and labeling protocol and GFX purification columns (Amersham Biosciences, Little Chalfont, Bucks., UK) were used. Together with blocking agents (12 μg of poly-(dA), 6 μg of yeast tRNA, and 20 μg of Cot-1 DNA), targets were hybridized to the microarrays for 18 hours under a glass coverslip with the use of humidified Corning hybridization chambers at 42°C and the Pronto Universal Hybridization System (Corning Inc., Corning, NY, USA). Slides were scanned at 10 μm resolution in an Agilent DNA Microarray Scanner (Agilent Technologies, Palo Alto, CA, USA) and the images were analyzed with GenePix Pro software (Axon Instruments, Union City, CA, USA).

### Microarray data analysis

The data were analyzed with BASE (BioArray Software Environment) software [30]. In brief, background-corrected intensities for sample and reference channels were calculated by subtracting the median local background signal from the median foreground signal for each spot. Filters were applied to remove all spots flagged during image analysis. Data within individual arrays were then normalized by using an implementation of the 'lowess' (locally weighted linear regression) algorithm [31]. Poorly measured/expressed spots with a signal-to-noise ratio of 3 or less in either the Cy3 or Cy5 channel were removed, and genes with missing data in more than 20% of all arrays or genes with a variation across arrays of not more than 0.45 standard deviations of the log_2_(ratio) were filtered, leaving 10,479 informative genes. The expression ratios for each gene were then median-centered across all tumors.

To generate a gene list for the basal-phenotype tumors, correlation scores were calculated between gene expression (log_2_(ratio)) for all reporters and the CK5/14 immunopositive tumors [32]. To evaluate the significance of the expression signatures between the two annotation classes (CK5/14-positive and CK5/14-negative), 1,000 permutations were run in which the samples were randomly given an annotation label, and the *p *value for a score was calculated as the average number of reporters exceeding the score in the permutation test, divided by the total number of reporters in the gene list. The false discovery rate – that is, the estimated number of genes in a given set of scored genes that could receive an equal or better score by chance – was calculated by random permutations and used as an indicator of the robustness of the gene expression profile. A false discovery rate of 0% indicates no false positives; a false discovery rate of 100% indicates a completely random signal. Gene expression profiles were analyzed with hierarchical clustering with centered Pearson correlation and average linkage clustering [33].

The ranked gene list was subjected to gene ontology annotation analysis with EASE (Expression Analysis Systematic Explorer) [34], in which only biological process ontology categories were included and the enrichment of categories in the gene list was evaluated by comparison with the total list of genes used for the microarray analysis. An EASE score of *p *≤ 0.05 was considered to be significant. The UniGene clusters representing the top 200 genes were annotated with subcellular location by cross-reference to two published microarray datasets [33,35] and to Swiss-Prot. The Swiss-Prot Subcellular Locations annotations were downloaded from the DRAGON database [36]. A gene was considered to be membrane associated or secreted if the Swiss-Prot annotation contained one of the words 'membrane', 'vesicle', or 'secreted', or if the membrane:cytosolic ratio in the polysome fraction study exceeded 2 or 1.08 in the studies by Diehn and colleagues [35] or Stitziel and colleagues [37], respectively. Primary expression data are available from the NCBI Gene Expression Omnibus database (accession ID GSE6768) [38].

## Results

### Immunohistochemical detection of basal-phenotype tumors

Immunohistochemical analysis was performed on TMAs containing 445 tumors, of which 375 (84%) were analyzable for CK5/CK14/p63 antibody cocktail. There were 48 (13%) CK5/14-positive and 13 (3.5%) p63-positive tumors. Although CK5/14 and p63 are co-expressed in normal cells of breast ducts, there was no association in malignant epithelial cells (*p *= 0.22). The CK5/14 immunopositivity was significantly correlated with negative ER status (*p *< 0.0001, data not shown). There were 13 ER-positive basal cytokeratin-expressing tumors. Association with negative progesterone receptor status (*p *< 0.0001) with negative lymph node status (*p *= 0.0005) and with p53 immunopositivity (*p *= 0.003) was also seen but there was no association with *HER-2 *oncogene amplification (*p *= 0.80, data not shown). Among the 95 ER-negative tumors, 35 (37%) showed positive staining for CK5/14 (Table 1). When CK5/14 positivity was correlated with clinicopathological characteristics within the ER-negative tumor subgroup, associations with negative lymph node status and positive p53 status were not seen (*p *= 0.14 and *p *= 0.65, respectively), but significant association between CK5/14 immunopositivity and negative *HER-2 *status emerged (*p *= 0.01, Table 1). Most of the basal cytokeratin-positive tumors were of the ductal histotype (80%) and the rest were of the medullary or atypical medullary histotypes (20%; Table 1). Over half (7/12) of the medullary histotype tumors (medullary or atypical medullary) were in fact CK5/14-positive.

### Gene expression profile of basal-phenotype tumors

**Figure 1:**
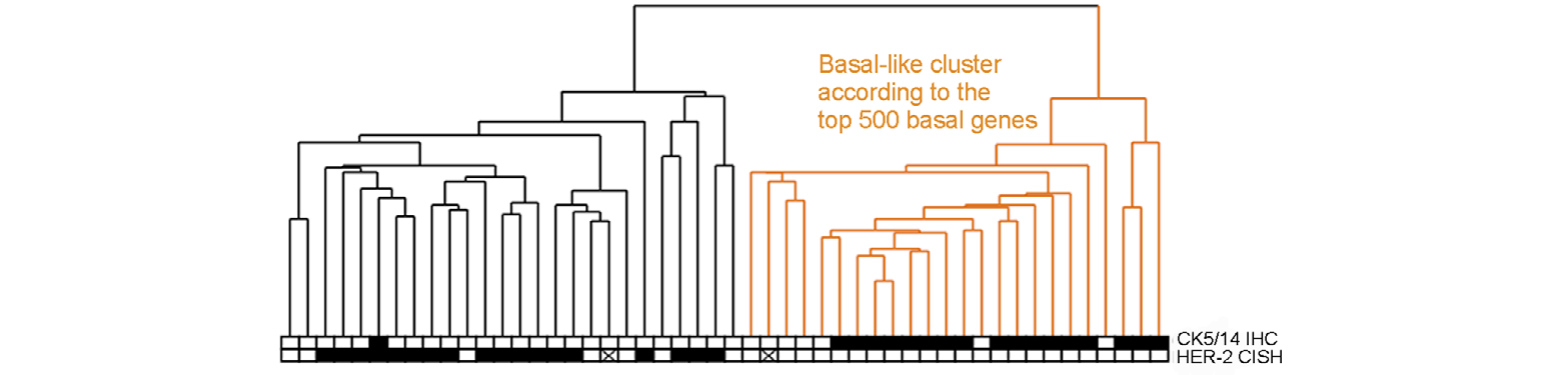
Hierarchical clustering of 50 ER-negative tumors based on the top 500 basal genes. The gene set was generated for the CK5/14-positive basal phenotype tumors. Yellow indicates the basal-like cluster and black the non-basal-like cluster. The boxes beneath indicate the immunohistochemically CK5/14-positive tumors and the *HER-2 *oncogene-amplified tumors (solid box, positive; open box, negative, crossed box, data missing).

A clear difference was seen in gene expression profiles between the basal cytokeratin (CK5/14) immunopositive and negative subgroups in the whole data set (false discovery rate 0.03% for the 100 genes, and 0.3% for the top 500 with the use of the Golub algorithm) including both ER-positive and ER-negative tumors. However, because the basal phenotype determined by IHC was strongly correlated with negative ER status (only one of the 50 ER-positive tumors stained positive for CK5/14), and because ER status has been shown to have a strong influence on the gene expression signature of breast tumors [2,4,6], we performed an analysis in the subset of ER-negative tumors (*n *= 50) separately. In this subset CK5/14-positive and CK5/14-negative tumors were also associated with two distinct gene expression signatures (false discovery rate 6.7% for the top 100 genes and 16.1% for the top 500 genes). Hierarchical clustering analysis of the ER-negative tumors using the top 500 basal discriminatory genes generated within the ER-negative tumor group identified two separate clusters (Figure 1; see Additional file 1 for the heat map): one cluster containing a large number of CK5/14-positive tumors (17/24) in addition to seven CK5/14-negative tumors, and another in which all except one of the tumors (25/26) were immunohistochemically CK5/14-negative and were frequently amplified for the *HER-2 *oncogene (18/26). Although the signal for the basal phenotype among ER-negative tumors was weaker than in the whole data set, in which the classification may have been highly influenced by the strong ER-related signal, it was statistically highly significant (1,713 genes were identified with *p *< 0.05; see Additional file 2 for the top 200 genes).

**Figure 2:**
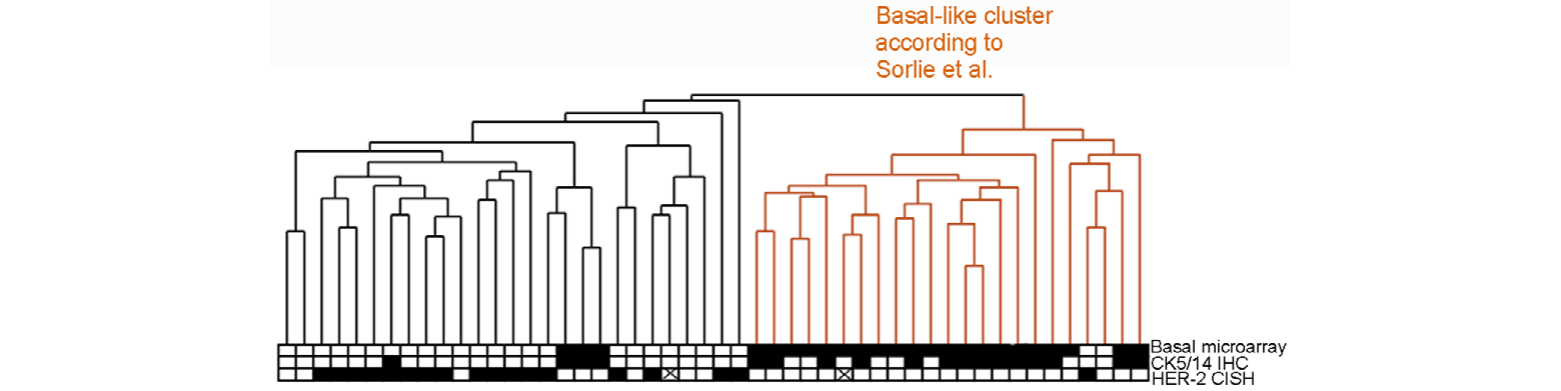
Hierarchical clustering of 50 ER-negative tumors based on the intrinsic gene set [7]. Yellow indicates the basal-like cluster and black the non-basal-like cluster. The black boxes beneath indicate the basal-like cluster by the top 500 basal genes, immunohistochemically CK5/14-positive tumors, and *HER-2 *amplified tumors (solid box, positive; open box, negative, crossed box, data missing).

We next explored how the so-called 'intrinsic' gene set generated by Perou and colleagues [6-8] would perform in our data set. Mapping of their intrinsic gene list [8] to our data with the use of Unigene Cluster ID as an identifier produced a list of 522 clones. These clones were used to cluster the whole data set, which gave expected results separating basal/ER-, luminal/ER+ and ERBB2+/ER- tumor groups from each other similarly to the original study (data not shown) [6,8]. Hierarchical clustering of the ER-negative tumor group separately, with the use of the intrinsic gene set, generated a dendrogram with two major subgroups very similar to the hierarchical clustering analysis with our top 500 ranked basal genes (concordance 90%, *p *= 0.0001; Figure 2). The basal-like cluster included most of the CK5/14-positive tumors and nine additional CK5/14-negative tumors. The tumors in the non-basal subgroup showed frequent *HER-2 *amplification (17/27) and predominantly a CK5/14-negative immunophenotype (23/27; Figure 2; see Additional file 3 for the heat map). The basal phenotype classification by Sorlie's intrinsic gene set correlated strongly with basal cytokeratin IHC (concordance 76%, *p *= 0.0011). Interestingly, seven of the nine misclassified CK5/14-negative tumors by Sorlie's intrinsic gene set were also found to belong to the basal-like cluster when our top 500 CK5/14-associated genes were used in hierarchical clustering analysis.

The gene list generated for the basal cytokeratin immunopositive tumors within the ER-negative tumor entity (Additional file 2) included genes associated with ER status such as *TTF1 *(rank 13) and *XBP1 *(rank 16) and other genes previously associated with the basal-like tumor subtype such as *CRYAB *(rank 26), *TRIM29 *(rank 51), *ERBB2 *(rank 55), *ANXA8 *(rank 134), and *EGFR *(rank 193) [6-9]. Twelve of the genes with a high expression in basal-like tumors (within the top 200 genes) were annotated as having a membrane-bound cellular localization, but not to the mitochondria or the Golgi apparatus (Additional file 2).

### Distant disease-free survival of basal-phenotype tumors

**Figure 3:**
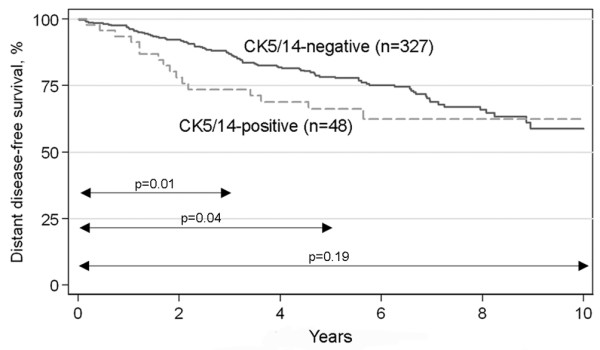
Distant disease-free survival of immunohistochemically CK5/14-negative and CK5/14-positive tumors in the whole data set. The basal cytokeratin-positive tumors show significantly shorter survival during the first years of the follow-up, but this difference is lost with time.

**Figure 4:**
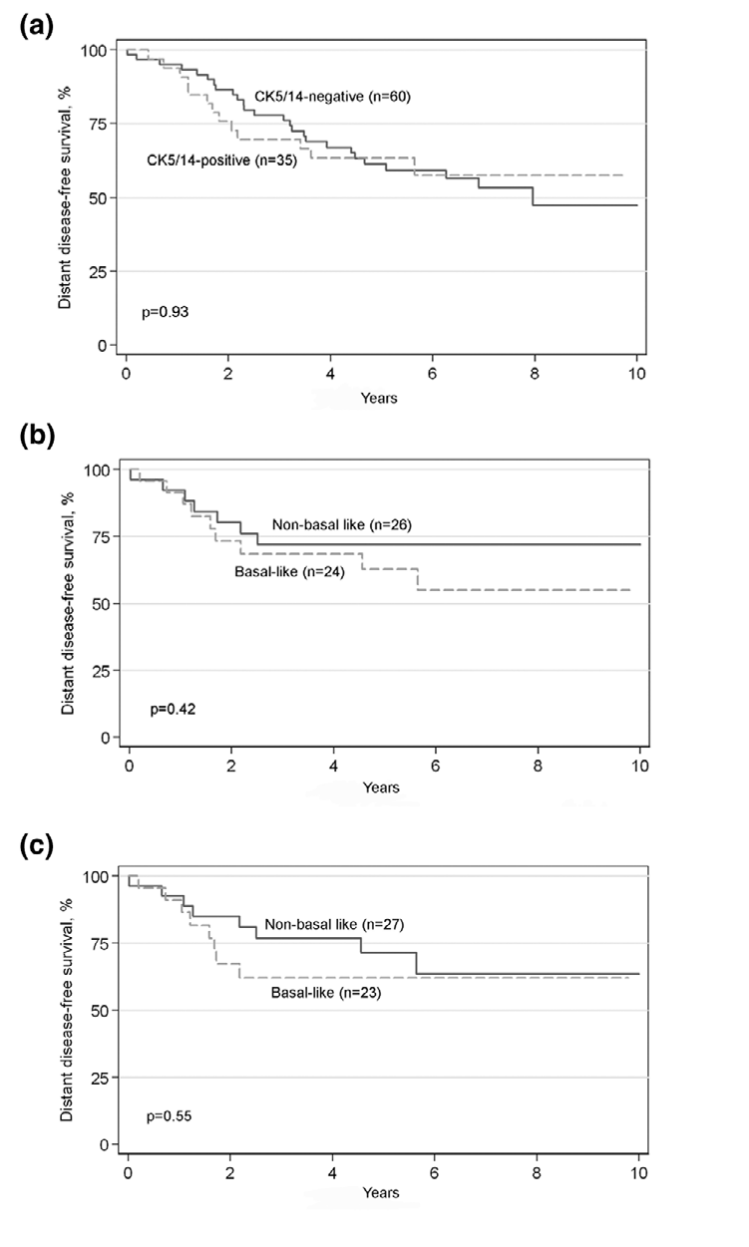
Distant disease-free survival of basal-like and non-basal-like tumors within the ER-negative tumor entity. The basal phenotype was defined by using immunohistochemistry **(a)**, cDNA microarray and the top 500 gene set for the basal cytokeratin-immunopositive tumors **(b) **or cDNA microarray and the intrinsic gene set of Sorlie and colleagues [7] **(c)**. There is no difference in survival between basal-like and non-basal-like tumors within the ER-negative tumor subgroup.

Association of the basal status with patient prognosis was evaluated first in the immunohistochemically defined basal (CK5/14-positive) and non-basal (CK5/14-negative) tumor subgroups. In the whole tumor material, the distant disease-free survival was significantly shorter for the CK5/14-positive tumors during the first years of follow-up (3 years hazard ratio (HR) 2.23, 95% confidence interval (CI) 1.17 to 4.24, *p *= 0.01 and 5 years HR 1.80, 95% CI 1.02 to 3.15, *p *= 0.04), but this difference was lost at the end of the follow-up period (10 years HR 1.43, 95% CI 0.84 to 2.43, *p *= 0.19; Figure 3). Next we studied clinical outcome within the ER-negative entity. The survival rates of immunohistochemically CK5/14-positive and CK5/14-negative tumor groups were identical, as demonstrated by the superimposed Kaplan-Meier curves and log-rank test (*p *= 0.93; Figure 4a). The same result was obtained when the basal-like classification was based on gene expression microarrays (*p *= 0.42 and *p *= 0.55 for classifications based on our gene list and Sorlie's gene list (Figure 4b,c), respectively).

### Functional analysis of genes aberrantly expressed in basal-phenotype tumors

We next performed a gene ontology (GO) annotation analysis of the top 1,000 genes on our basal gene list (within ER-negative tumors) and found that 823 genes were associated with a functional gene annotation category. Of these genes, 383 were upregulated in the CK5/14-positive tumors and 440 were downregulated (Additional file 4). Genes upregulated in basal-like tumors (with an EASE score of 0.05 or less) belonged to the annotation categories epidermal differentiation (GO:0008544) and ectoderm development (GO:0007398), protein biosynthesis (GO:0006412), nuclear division (GO:0000280), development (GO:0007275), biosynthesis (GO:0009058), histogenesis (GO:0009888), macromolecule biosynthesis (GO:0009059), and M phase (GO:0000279). Basal cytokeratins 14 and 17 were present in the gene category of epidermal differentiation and ectoderm development, which was the most significantly upregulated biological process in basal-phenotype tumors. Genes downregulated in basal-phenotype tumors were characterized as having functions in cell-surface receptor-linked signal transduction (GO:0007166), enzyme-linked receptor protein signaling pathway (GO:0007167), transmembrane receptor protein tyrosine kinase signaling pathway (GO:0007169), and regulation of G-protein-coupled receptor protein signaling pathway (GO:0008277).

## Discussion

Basal-like breast cancer has been associated with poor prognosis in several immunohistochemical [10,11,15-18,20,22-25] and gene expression microarray-based studies [7-9]. Nevertheless, there are conflicting results between studies about the independent prognostic significance of the basal phenotype [11,15,18,20]. Adjuvant chemotherapy could be recognized as one possible confounding factor, because it has been postulated that basal-like and non-basal tumors would respond differently to chemotherapy [39]. Our results showed that when using IHC to identify basal-like tumors, a survival difference was seen in the entire patient population during the first years of the follow-up. This suggests that basal cytokeratin expression predicts early relapse when compared with non-basal tumors, including both ER-positive and ER-negative breast cancers. This is in agreement with previous results [11,15-18,20,22-25]. Furthermore, our tumor series represents early-stage disease not treated with chemotherapy. It therefore presents a more coherent picture of the natural biology of breast cancer than when studying chemotherapy-treated patients. It must still be noted that in this study all the patients were treated with tamoxifen for 2 years, which most probably affected the natural history of the ER-positive tumors.

Even though we saw a survival difference between basal and non-basal tumors when studying the whole population, this was not true within the ER-negative tumor subgroup. This therefore suggests that basal cytokeratin expression is not an independent prognostic factor. Our results support the findings of Potemski and colleagues [18] and Malzahn and colleagues [15], who did not find any difference between basal and non-basal tumor survival within the ER-negative tumor entity. However, Abd El-Rehim and colleagues [11] and Rakha and colleagues [20] have suggested that adjustment to steroid hormone receptor expression would not alter the adverse survival impact of basal phenotype in breast cancer. In our study the lack of prognostic association was not due to the method of tumor classification, because the same result was obtained within the ER-negative subgroup when basal-like tumors were identified either by IHC or by two different microarray-based classifications. These results are in agreement with the earlier microarray-based prognostic studies, which indicate that tumors with a basal-like gene expression signature have a similar prognosis to that of the ERBB2 cluster [7-9]. It is concluded that all ER-negative tumors can be classified as having a relatively poor prognosis, irrespective of the cytokeratin composition or gene expression signature.

Studies of basal-like breast cancer are likely to be influenced by the ER status, which is a central factor determining both prognosis and gene expression patterns [1,2,5,6]. To study the basal-phenotype breast cancer more specifically without the influence of ER status, we performed a gene expression microarray study for ER-negative breast cancers. This enabled us to look more specifically at the gene expression profile and clinical behavior of the basal-phenotype tumors when the impact of information already included in the ER status was excluded. We were able to separate two tumor clusters, the basal-like and the non-basal-like, by using a gene set generated for the basal cytokeratin immunopositive tumors. The unique gene expression profile found for the CK5/14 immunopositive tumors within the ER-negative tumor entity implies that the basal-like expression profile differed significantly from the rest of the ER-negative tumors and that this tumor subgroup is biologically distinct not only in the general breast cancer population but also within ER-negative tumor entity.

Our CK5/14-associated gene signature identified basal-like tumors within the ER-negative tumor entity very similarly to the clustering with the intrinsic gene set by Sorlie and colleagues [7]. Whereas all except one of the CK5/14-positive tumors were classified to the basal-like cluster with our CK5/14-associated genes, four tumors with a CK5/14-positive immunophenotype were found in the non-basal-like cluster with Sorlie's intrinsic gene set. This indicates that our top 500 ranked basal genes were better classifiers for CK5/14 IHC status than Sorlie's intrinsic gene set. This is not surprising given that our basal gene list was generated for this purpose and from this very material. Interestingly, all seven CK5/14-negative tumors categorized into the basal-like cluster by our basal-associated genes were also found in the basal-like tumor subgroup when performing the analysis with the intrinsic gene set as defined by Sorlie and colleagues. Hence, for these seven cases the two microarray-analysis-based classifiers agreed on the basal-like status but disagreed with the CK5/14 immunostaining.

To verify that these tumors had not been misclassified with regard to basal-like status when using TMAs, we immunostained the entire tumor sections of five of these tumors. Two of the tumors were scored as CK5/14 positive in entire sections, indicating that the TMA sampling technique (using tissue cores with 0.6 mm diameter) leads to the misclassification of some basal-like tumors in IHC. Expression of basal cytokeratins often shows a high degree of intratumoral heterogeneity [22], which is likely to explain differences obtained between TMAs and entire tissue sections. However, even when performed on entire tumor sections, CK5/14 IHC may not recognize all of the basal-like subtype breast cancers as defined by gene expression profiles. Despite the fact that our gene expression signature was generated to be specifically associated with CK5/14 positivity, it clearly also recognizes a distinct set of CK5/14-negative tumors.

It has previously been suggested that the basal-like tumor type cluster is most optimally identified by IHC when using a combination of positive CK5/6 and/or EGFR, and negative ER and HER-2 staining results as classification criteria [23,40]. In addition, vimentin and c-*kit*, which have been shown to be associated with basal cytokeratin immunopositivity along with EGFR [22,41], have been recognized as good discriminators for a basal-like expression profile [23,40]. The basal cytokeratin-negative tumors that clustered with the basal-like cluster in this study could be EGFR, vimentin, and/or c-*kit*-expressing tumors with a similar gene expression signature to that of basal cytokeratin-immunopositive breast cancers. It is concluded that immunohistochemically basal cytokeratin-positive tumors almost always belong to the basal-like gene expression profile, but this cluster also includes basal cytokeratin-negative tumors. Neither a immunohistochemical nor a microarray-based classification of breast cancers into a basal or non-basal subgroup is currently considered justified in the clinics, because direct predictive or prognostic implications are lacking. This could change in the future if differential treatment responsiveness could be confirmed or if treatments specifically targeting basal-like tumors were developed.

In addition to prognostic assessments, the microarray-based gene data may be more relevant for revealing the biological basis of the basal-like tumor classification. For example, the first genes in the gene list generated for the immunohistochemically predefined CK5/14-positive and ER-negative tumors included some genes, such as *XBP1 *and *TTF1*, that are known to associate positively with ER status [1,2,6]. These genes had a significantly lower expression in the basal-like than in the non-basal-like tumors within the ER-negative tumor subgroup. It is therefore possible that there are some differences in the hormone-independence of the basal-like and non-basal-like tumors within the ER-negative tumor subgroup. In addition to ER-negativity and poor response to hormone treatment, most basal-like tumors are *HER-2 *non-amplified. There are therefore currently no targeted treatment options available for basal-like breast cancers. Our finding that top signature genes such as *EVA1 *(rank 11 and 36), *SLC2A1 *(rank 42 and 179), and *CEACAM1 *(rank 148), which are highly expressed in basal-like tumors and are localized to the cell membrane, could serve as interesting targets for new drug developments, similar to the HER-2 oncoprotein in tumors with *ERBB2 *gene amplification.

To study the biology of basal-like tumors in more detail and to evaluate the function of the genes found associated with this tumor subtype we next found out which biological processes were enriched in basal-like tumors and used EASE for this purpose. We found that the signature for basal-like tumors was most significantly enriched for genes associated with epidermal differentiation and included the genes encoding CK14 and CK17. Both of these cytokeratins are close partners of CK5 [42] and have been shown to be expressed in basal-phenotype tumors by IHC [11,12,17,20] and by gene expression microarrays [6,7]. We did not use CK17 in the immunohistochemical determination of basal cytokeratin expression because we had shown previously that only very few tumors show CK17 expression in the absence of CK5 and/or CK14 [12]. The biological process of epidermal differentiation may reflect the basal-phenotype tumor origin. It has been suggested that a CK5/14-positive breast progenitor cell able to differentiate into both luminal and myoepithelial cells of the normal breast would be the transformed cell in basal-phenotype breast cancer [43,44]. If these cells represent the so-called cancer stem cell for basal-phenotype breast cancer, the tumor cells may have the same ability to differentiate as the cell of origin does. The biological process of development was fourth in the ranking list and included the *EVA1 *gene, which was previously recognized in the basal gene list (rank 11 and 36) as a membrane protein. Other gene ontology terms enriched in the basal-like gene signature, such as protein and macromolecular biosynthesis, nuclear division, and M phase, were indicative of a high proliferation rate. Previous studies have also associated the basal-like subgroup with a high expression of genes involved in proliferation [14,22], and our results suggest that this is true even when compared with the other subgroups, such as amplified *HER-2*, within the ER-negative entity.

## Conclusion

Basal cytokeratin immunopositivity predicts early breast cancer relapse, and these tumors differ from other ER-negative breast cancers biologically because they have a distinct gene expression profile. Despite this, the basal cytokeratin-expressing tumors show a similar prognosis to that of non-basal ER-negative tumors. As regards classification, immunohistochemically basal cytokeratin-positive tumors almost always show a basal-like gene expression signature. We were able to identify several immunohistochemically basal cytokeratin-negative tumors, which have a similar gene expression profile to that of the basal cytokeratin-immunopositive breast cancers.

## Abbreviations

CI = confidence interval; CISH = chromogenic *in situ *hybridization; EASE = Expression Analysis Systematic Explorer; EGFR = epidermal growth factor receptor; ER = estrogen receptor; GO = gene ontology; HR = hazard ratio; IHC = immunohistochemistry; TMA = tissue microarray.

## Competing interests

The authors declare that they have no competing interests.

## Authors' contributions

MJ performed and analyzed IHC and CISH stainings from the TMAs, and drafted and finalized the manuscript. SG performed and analyzed the microarrays and helped in the drafting of the manuscript. Päivikki Kauraniemi helped with the interpretation of the results and with drafting the manuscript. MT helped with the finalization of the manuscript. PB performed the statistics for the tables and figures. MK conducted the analysis of the membrane association of the genes. Pasi Kataja performed the scanning of the slides for virtual microscopy, and ML prepared the final virtual slides for the Internet. ÅB and MF coordinated the study on their behalf. JI coordinated the study and helped to draft and finalize the manuscript. All authors read and approved the final manuscript.

## Supplementary Material

Additional File 1 A PDF file containing a heat map of 50 ER-negative tumors based on the top 500 gene set generated for the CK5/14-positive tumors. Yellow indicates the basal-like cluster and black the non-basal-like cluster. Click here for file

Additional File 2 An Excel file containing the top 200 genes list generated for the immunohistochemically CK5/14-positive ER-negative breast cancers. The membrane association is defined. Click here for file

Additional File 3 A PDF file containing a heat map of 50 ER-negative tumors based on the intrinsic gene set by Sorlie and colleagues [7]. Yellow indicates the basal-like cluster and black the non-basal-like cluster. Click here for file

Additional File 4 A PDF file containing the results of a gene ontology annotation analysis of the top 1,000 basal genes. Click here for file

## Figures and Tables

**Table 1:** Clinicopathological characteristics of estrogen-receptor-negative breast tumors according to basal cytokeratin (CK5/14) status

Clinicopathological parameter	CK5/14 negative (percentage)	CK5/14 positive (percentage)	*p*
All estrogen-receptor-negative tumors	60 (63)	35 (37)	
Axillary lymph node status			
Negative	23 (38)	19 (54)	0.14
Positive	37 (62)	16 (46)	
*HER-2 *status			
Non-amplified	35 (58)	30 (86)	0.01
Amplified	18 (30)	3 (9)	
Data missing	7 (12)	2 (6)	
p63			
Negative	57 (95)	33 (94)	1.00
Positive	3 (5)	2 (6)	
p53			
Negative	26 (43)	13 (37)	0.65
Positive	26 (43)	17 (49)	
Data missing	8 (13)	5 (14)	
Histological type			
Invasive ductal or mixed type	49 (82)	28 (80)	0.10
Invasive lobular	5 (8)	0 (0)	
Medullary or atypical medullary	5 (8)	7 (20)	
Other types	1 (2)	0 (0)	

The *p *values were calculated, excluding the 'data missing' values, with Fisher's exact test except for the *p *value of histological type, which was calculated with the χ^2 ^test.
