# Supplementary figures and images for: Basal-like phenotype is not associated with patient survival in estrogen-receptor-negative breast cancers
Source: Breast Cancer Res. 2007 Jan 31;9(1):R16. doi: 10.1186/bcr1649 (PMC1851391; doi:10.1186/bcr1649)

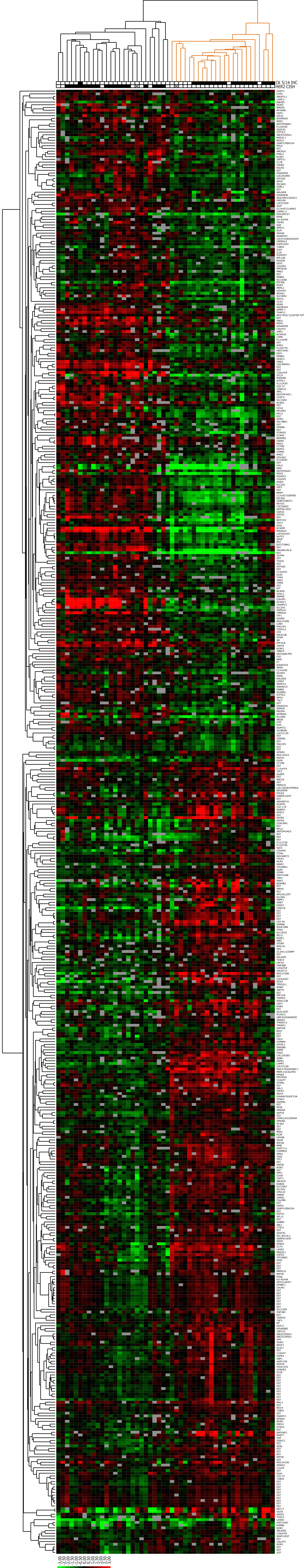

Supplement: Additional File 1 — A PDF file containing a heat map of 50 ER-negative tumors based on the top 500 gene set generated for the CK5/14-positive tumors. Yellow indicates the basal-like cluster and black the non-basal-like cluster. [file bcr1649-S1.pdf]

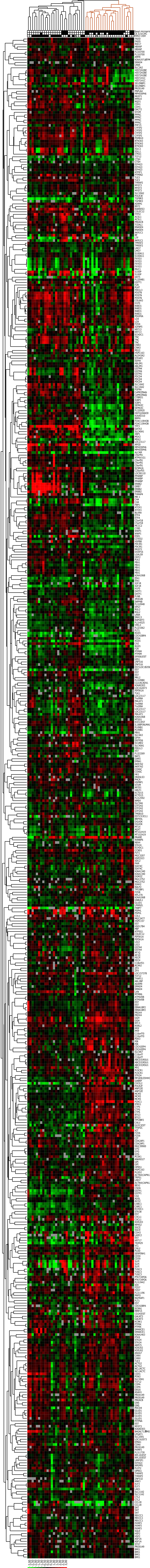

Supplement: Additional File 3 — A PDF file containing a heat map of 50 ER-negative tumors based on the intrinsic gene set by Sorlie and colleagues [7]. Yellow indicates the basal-like cluster and black the non-basal-like cluster. [file bcr1649-S3.pdf]
