# Supplementary material for: Basal-like phenotype is not associated with patient survival in estrogen-receptor-negative breast cancers
Source: Breast Cancer Res. 2007 Jan 31;9(1):R16. doi: 10.1186/bcr1649 (PMC1851391; doi:10.1186/bcr1649)
Supplement: Additional File 4 — A PDF file containing the results of a gene ontology annotation analysis of the top 1,000 basal genes. [file bcr1649-S4.pdf]

	Gene Category	EASE score	Gene Rank	Gene Name	Gene Symbol	Unigene ID
**Up-regulated in CK5/14+**	Epidermal differentiation & Ectoderm development	0.007	329	Keratin 17	KRT17	HS.2785
	Biological Process GO:0008544 & GO:0007398		868	Keratin 14 (epidermolysis bullosa simplex, Dowling-Meara, Koebne	KRT14	HS.355214
			828	Fatty acid binding protein 5 (psoriasis-associated)	FABP5	HS.408061
			92	Desmoplakin	DSP	HS.519873
			664	Procollagen-lysine 1, 2-oxoglutarate 5-dioxygenase (lysine hydroxy	PLOD	HS.75093
	Protein biosynthesis	0.027	276	UDP-GlcNAc:betaGal beta-1,3-N-acetylglucosaminyltransferase 5	B3GNT5	HS.257222
	Biological Process GO:0006412		870	Alanyl-tRNA synthetase	AARS	HS.315137
			778	Ribosomal protein S5	RPS5	HS.378103
			865	Eukaryotic translation initiation factor (eIF) 2A	eIF2A	HS.378808
			333	Mitochondrial ribosomal protein S10	MRPS10	HS.380887
			442	Eukaryotic translation initiation factor 3, subunit 6 48kDa	EIF3S6	HS.405590
			934	Ribosomal protein S10	RPS10	HS.406620
			615	Prostaglandin F2 receptor negative regulator	PTGFRN	HS.418093
			433	KIAA0056 protein	KIAA0056	HS.438550
			630	Chromosome 1 open reading frame 33	C1orf33	HS.463797
			131	ElaC homolog 1 (E. coli)	ELAC1	HS.47572
			408	Mitochondrial ribosomal protein L2	MRPL2	HS.55041
			522	Eukaryotic translation elongation factor 1 epsilon 1	EEF1E1	HS.88977
			470	Ribonuclease P/MRP 38kDa subunit	RPP38	HS.94986
	Nuclear division	0.031	930	Cell division cycle 25A	CDC25A	HS.1634
	Biological Process GO:0000280		791	Cyclin B2	CCNB2	HS.194698
			633	Cyclin B1	CCNB1	HS.23960
			646	CHK1 checkpoint homolog (S. pombe)	CHEK1	HS.24529
			939	SKB1 homolog (S. pombe)	SKB1	HS.367854
			577	RAD51 homolog (RecA homolog, E. coli) (S. cerevisiae)	RAD51	HS.446554
			285	Kinesin family member 2C	KIF2C	HS.69360
			158	Cyclin A2	CCNA2	HS.85137
	Development	0.033	5	Jerky homolog-like (mouse)	JRKL	HS.105940
	Biological Process GO:0007275		11	Epithelial V-like antigen 1	EVA1	HS.116651
			504	zinc finger protein 74 (Cos52)	ZNF74	HS.127476
			34	Neighbor of COX4	NOC4	HS.173162
			197	PAP associated domain containing 1	PAPD1	HS.173946
			710	Dynamin 1-like	DNM1L	HS.180628
			824	High-mobility group box 3	HMGB3	HS.19114
			574	Bridging integrator 1	BIN1	HS.193163
			755	SRY (sex determining region Y)-box 9 (campomelic dysplasia, autc	SOX9	HS.2316
			646	CHK1 checkpoint homolog (S. pombe)	CHEK1	HS.24529
			276	UDP-GlcNAc:betaGal beta-1,3-N-acetylglucosaminyltransferase 5	B3GNT5	HS.257222
			165	UDP glycosyltransferase 8 (UDP-galactose ceramide galactosyltrar	UGT8	HS.274293
			329	Keratin 17	KRT17	HS.2785
			390	Alpha-2-HS-glycoprotein	AHSG	HS.324746
			305	Forkhead box C1	FOXC1	HS.348883
			868	Keratin 14 (epidermolysis bullosa simplex, Dowling-Meara, Koebne	KRT14	HS.355214
			828	Fatty acid binding protein 5 (psoriasis-associated)	FABP5	HS.408061
			26	Crystallin, alpha B	CRYAB	HS.408767
			835	kinesin family member 1B	KIF1B	HS.444757
			550	Glutaredoxin 2	GLRX2	HS.458283
			92	Desmoplakin	DSP	HS.519873
			513	Chaperonin containing TCP1, subunit 6B (zeta 2)	CCT6B	HS.73072
			637	T-cell acute lymphocytic leukemia 1	TAL1	HS.73828
			664	Procollagen-lysine 1, 2-oxoglutarate 5-dioxygenase (lysine hydroxy	PLOD	HS.75093
			465	Carboxypeptidase Z	CPZ	HS.78068
			33	Kallikrein 6 (neurosin, zyme)	KLK6	HS.79361
			50	Chromosome 4 open reading frame 14	C4orf14	HS.8715
	Biosynthesis	0.035	748	Hypothetical protein CL640	CL640	HS.144304
	Biological Process GO:0009058		3	Adenosylmethionine decarboxylase 1	AMD1	HS.159118
			276	UDP-GlcNAc:betaGal beta-1,3-N-acetylglucosaminyltransferase 5	B3GNT5	HS.257222
			165	UDP glycosyltransferase 8 (UDP-galactose ceramide galactosyltrar	UGT8	HS.274293
			204	Cytidine monophosphate N-acetylneuraminic acid synthetase	CMAS	HS.311346
			870	Alanyl-tRNA synthetase	AARS	HS.315137
			778	Ribosomal protein S5	RPS5	HS.378103
			865	Eukaryotic translation initiation factor (eIF) 2A	eIF2A	HS.378808
			281	stearoyl-CoA desaturase 4	SCD4	HS.379191
			333	Mitochondrial ribosomal protein S10	MRPS10	HS.380887
			442	Eukaryotic translation initiation factor 3, subunit 6 48kDa	EIF3S6	HS.405590
			934	Ribosomal protein S10	RPS10	HS.406620
			615	Prostaglandin F2 receptor negative regulator	PTGFRN	HS.418093
			887	Methylenetetrahydrofolate dehydrogenase (NADP+ dependent) 1, r	MTHFD1	HS.435974
			433	KIAA0056 protein	KIAA0056	HS.438550
			417	Uridine-cytidine kinase 2	UCK2	HS.458360
			630	Chromosome 1 open reading frame 33	C1orf33	HS.463797
			131	ElaC homolog 1 (E. coli)	ELAC1	HS.47572
			408	Mitochondrial ribosomal protein L2	MRPL2	HS.55041
			394	Adenylosuccinate lyase	ADSL	HS.75527
			522	Eukaryotic translation elongation factor 1 epsilon 1	EEF1E1	HS.88977
			470	Ribonuclease P/MRP 38kDa subunit	RPP38	HS.94986
	Histogenesis	0.037	461	CD59 antigen p18-20 (antigen identified by monoclonal antibodies 1	CD59	HS.2785
	Biological Process GO:0009888		868	Keratin 14 (epidermolysis bullosa simplex, Dowling-Meara, Koebne	KRT14	HS.355214
			828	Fatty acid binding protein 5 (psoriasis-associated)	FABP5	HS.408061
			92	Desmoplakin	DSP	HS.519873
			664	Procollagen-lysine 1, 2-oxoglutarate 5-dioxygenase (lysine hydroxy	PLOD	HS.75093
	Macromolecule biosynthesis	0.041	276	UDP-GlcNAc:betaGal beta-1,3-N-acetylglucosaminyltransferase 5	B3GNT5	HS.257222
	Biological Process GO:0009059		165	UDP glycosyltransferase 8 (UDP-galactose ceramide galactosyltrar	UGT8	HS.274293
			204	Cytidine monophosphate N-acetylneuraminic acid synthetase	CMAS	HS.311346
			870	Alanyl-tRNA synthetase	AARS	HS.315137
			778	Ribosomal protein S5	RPS5	HS.378103
			865	Eukaryotic translation initiation factor (eIF) 2A	eIF2A	HS.378808
			281	stearoyl-CoA desaturase 4	SCD4	HS.379191
			333	Mitochondrial ribosomal protein S10	MRPS10	HS.380887
			442	Eukaryotic translation initiation factor 3, subunit 6 48kDa	EIF3S6	HS.405590
			934	Ribosomal protein S10	RPS10	HS.406620
			615	Prostaglandin F2 receptor negative regulator	PTGFRN	HS.418093
			887	Methylenetetrahydrofolate dehydrogenase (NADP+ dependent) 1, r	MTHFD1	HS.435974
			433	KIAA0056 protein	KIAA0056	HS.438550
			630	Chromosome 1 open reading frame 33	C1orf33	HS.463797
			131	ElaC homolog 1 (E. coli)	ELAC1	HS.47572
			408	Mitochondrial ribosomal protein L2	MRPL2	HS.55041
			394	Adenylosuccinate lyase	ADSL	HS.75527
			522	Eukaryotic translation elongation factor 1 epsilon 1	EEF1E1	HS.88977
			470	Ribonuclease P/MRP 38kDa subunit	RPP38	HS.94986
	M phase	0.044	470	Ribonuclease P/MRP 38kDa subunit	RPP38	HS.1634
	Biological Process GO:0000279		791	Cyclin B2	CCNB2	HS.194698
			633	Cyclin B1	CCNB1	HS.23960
			646	CHK1 checkpoint homolog (S. pombe)	CHEK1	HS.24529
			939	SKB1 homolog (S. pombe)	SKB1	HS.367854
			577	RAD51 homolog (RecA homolog, E. coli) (S. cerevisiae)	RAD51	HS.446554
			285	Kinesin family member 2C	KIF2C	HS.69360
			158	Cyclin A2	CCNA2	HS.85137
**Down-regulated in CK5/14+**	Cell surface receptor linked signal transduction	<0.001	452	Ral guanine nucleotide dissociation stimulator	RALGDS	HS.106185
	Biological Process GO:0007166		463	Transforming growth factor, beta 1 (Camurati-Engelmann disease)	TGFB1	HS.1103
			845	Calcium and integrin binding 1 (calmyrin)	CIB1	HS.135471
			443	Fibroblast growth factor receptor 4	FGFR4	HS.165950
			291	Interferon-stimulated transcription factor 3, gamma 48kDa	ISGF3G	HS.1706
			512	Integrin, alpha L	ITGAL	HS.174103
			428	Adenosine A2a receptor	ADORA2A	HS.197029
			438	Regulator of G-protein signalling 5	RGS5	HS.24950
			801	Transducin-like enhancer of split 3 (E(sp1) homolog, Drosophila)	TLE3	HS.287362
			396	Regulator of G-protein signalling 16	RGS16	HS.413297
			568	Sprouty homolog 1, antagonist of FGF signaling (Drosophila)	SPRY1	HS.436944
			55	V-erb-b2 erythroblastic leukemia viral oncogene homolog 2	ERBB2	HS.446352
			189	Regulator of G-protein signalling 11	RGS11	HS.65756
			15	Bone morphogenetic protein 4	BMP4	HS.68879
			923	GRB2-associated binding protein 1	GAB1	HS.80720
			679	MAP-kinase activating death domain	MADD	HS.82548
			309	Growth factor receptor-bound protein 7	GRB7	HS.86859
	Enzyme linked receptor protein signaling pathway	0.002	463	Transforming growth factor, beta 1 (Camurati-Engelmann disease)	TGFB1	HS.1103
	Biological Process GO:0007167		443	Fibroblast growth factor receptor 4	FGFR4	HS.165950
			568	Sprouty homolog 1, antagonist of FGF signaling (Drosophila)	SPRY1	HS.436944
			55	V-erb-b2 erythroblastic leukemia viral oncogene homolog 2	ERBB2	HS.446352
			15	Bone morphogenetic protein 4	BMP4	HS.68879
			923	GRB2-associated binding protein 1	GAB1	HS.80720
			309	Growth factor receptor-bound protein 7	GRB7	HS.86859
	Transmembrane receptor protein tyrosine kinase signaling pathway	0.018	443	Fibroblast growth factor receptor 4	FGFR4	HS.165950
	Biological Process GO:0007169		568	Sprouty homolog 1, antagonist of FGF signaling (Drosophila)	SPRY1	HS.436944
			55	V-erb-b2 erythroblastic leukemia viral oncogene homolog 2	ERBB2	HS.446352
			923	GRB2-associated binding protein 1	GAB1	HS.80720
			309	Growth factor receptor-bound protein 7	GRB7	HS.86859
	Regulation of G-protein coupled receptor protein signaling pathway	0.029	438	Regulator of G-protein signalling 5	RGS5	HS.24950
	Biological Process GO:0008277		396	Regulator of G-protein signalling 16	RGS16	HS.413297
			189	Regulator of G-protein signalling 11	RGS11	HS.65756
